# Rapid and Simple Detection of Viable Foodborne Pathogen *Staphylococcus aureus*

**DOI:** 10.3389/fchem.2019.00124

**Published:** 2019-03-12

**Authors:** Caiyan Liu, Chao Shi, Mengzhe Li, Mengyuan Wang, Cuiping Ma, Zonghua Wang

**Affiliations:** ^1^Shandong Sino-Japanese Center for Collaborative Research of Carbon Nanomaterials, College of Chemistry and Chemical Engineering, College of Life Sciences, Qingdao University, Qingdao, China; ^2^The Affiliated Hospital of Qingdao University Medical College, Qingdao, China; ^3^Shandong Provincial Key Laboratory of Biochemical Engineering, College of Marine Science and Biological Engineering, Qingdao University of Science and Technology, Qingdao, China

**Keywords:** strand exchange amplification, isothermal amplification, *Staphylococcus aureus*, rapid detection, point-of-care testing

## Abstract

*Staphylococcus aureus* (*S. aureus*) contamination in food safety has become a worldwide health problem. In this work, we utilized RNA one-step detection of denaturation bubble-mediated Strand Exchange Amplification (SEA) method to realize the detection of viable foodborne pathogen *S. aureus*. A pair of *S. aureus* specific primers were designed for the SEA reaction by targeting hypervariable V2 region of 16S rDNA and the amplification reaction was finished about 1 h. The results of amplification reaction could be observed by the naked eyes with a significant color change from light yellow to red to realize the colorimetric detection of *S. aureus*. Therefore, there only required an isothermal water bath, which was very popular for areas with limited resources. In real sample testing, although the SEA detection was so time-saving compared with the traditional plating method, the SEA method showed great consistency with the traditional plating method. In view of the above-described advantages, we provided a simple, rapid and equipment-free detection method, which had a great potential on ponit-of-care testing (POCT) application. Our method reported here will also provide a POCT detection platform for other food-borne pathogens in food, even pathogenic bacteria from other fields.

## Introduction

*Staphylococcus aureus* (*S. aureus*), a facultative anaerobic Gram-positive coccus, can greatly threaten our health due to a combination of toxin-mediated virulence, invasiveness, and antibiotic resistance (Oliveira et al., 2011). For example, *S. aureus* is the main cause of nosocomial infections and community-acquired diseases, including deep-seated, endocarditis, abscesses and bacteria, which lead to toxic and septic shock syndromes (Abdalhai et al., 2014). *S. aureus* widely exists in the air, water, dust, human and animal excretions, which makes the food much easier to become contaminated (Yu et al., 2016). It has been reported that *S. aureus* contamination is a worldwide health problem. In the United States, nearly half a million hospitalizations and 50,000 deaths occur resulting from *S. aureus* each year (Schlecht et al., 2015). Likewise, 11 outbreaks of *Staphylococcal* food poisoning were reported between 2006 and 2009 in Shenzhen, China, which ranked the second most frequent cause of bacterial food poisoning (Yan et al., 2012). Therefore, an efficient *S. aureus* detection method is necessary for food safety and human health.

To date, *S. aureus* has been listed as a legal testing item in food safety of most countries worldwide (Xiong et al., 2016). The conventional *S. aureus* detection method is performed by the culture-based technique, which is time-consuming (4–7 days) (Roda et al., 2012). Immunological procedures, including immunoprecipitation, enzyme-labeled immunosorbent assay, and immunoblotting are also used for detecting *S. aureus* based on specific binding of antigen and antibody (Min et al., 2011). However, the sensitivity of these methods are low and their operations are usually very difficult and tedious, which make them hard to popularize (Kimura et al., 2013). Recently, with the development of molecular biology technology, polymerase chain reaction (PCR) (Niraj et al., 2016) has been widely used for the *S. aureus* nucleic acid detection. These methods derived from PCR require sophisticated instruments and lifting temperature, and they are not suitable for the point-of-care (POCT) testing. Considering the wide distribution and the harmful effects of *S. aureus*, therefore, a rapid and simple method for the detection of *S. aureus* was seriously required. Recently, a strand exchange amplification (SEA), mediated by denaturation bubbles, was established by Shi et al. (2016). Similarly to the traditional PCR technique, SEA employed a *Bst* DNA polymerase and a pair of primers to carry out an exponential DNA amplification under an isothermal condition. In addition, *Bst* DNA polymerase has intrinsic reverse transcriptase activity (Shi et al., 2015), which makes the SEA method able to detect RNA directly without additional reverse transcription. Therefore, in this work, we firstly utilized RNA one-step detection of SEA method to realize the detection of viable foodborne pathogen *S. aureus*. In particular, the sensitivity of SEA method was greatly improved in this work due to the high abundance of RNA in live bacteria. The whole SEA detection procedure for real samples took only 1–2 h. In addition, the colorimetric detection of *S. aureus* was also developed to realize the detection result read out by the naked eyes. We firstly utilized RNA one-step detection of SEA method to realize the detection of viable foodborne pathogen *S. aureus*. The sensitivity of SEA method especially, was greatly improved in this work due to the high abundance of RNA in live bacteria. The whole SEA detection procedure for real samples took only 1–2 h. In addition, the colorimetric detection of *S. aureus* was also developed to realize the detection result read out by the naked eyes. Therefore, there was only the need for a metal bath, which was very popular for areas with limited resources. In brief, we firstly used the SEA method for rapid and simple detection of foodborne pathogen *S. aureus*, even viable *S. aureus*, to further ensure food safety.

## Materials and Methods

### Reagents and Bacterial Strains

SEA detection kits were obtained from Qingdao Navid Biotechnology Co. Ltd. (China). Twenty bp DNA marker, 6 × DNA loading buffer and sodium dodecyl sulfate (SDS) were purchased from Sangon Biotech (Shanghai, China). Acrylamide and methylene diacrylamide were purchased from Sigma-Aldrich (St Louis, MO, USA). Recombinant DNase I was purchased from Takara Bio (Beijing, China). RNA-Be-Gone was purchased from Sangon Biotech (Shanghai, China). The bacterial strains including *S. aureus, Listeria monocytogenes (L. monocytogenes), Salmonella typhimurium (S. typhimurium), Shigella castellani (S. castellani), Vibrio parahemolyticus (V. parahemolyticus)*, and *Escherichia coli (E. coli)* were stored in our laboratory.

### Primers Design and Synthesis

A pair of specific primers (Table 1) were designed in the hypervariable region of *S. aureus* 16S rDNA with the NUPACK software (http://www.nupack.org/) and DINAMelt Web Server (http://unafold.rna.albany.edu/?q=DINAMelt). The primers were synthesized and purified by high performance liquid chromatography (HPLC) in Sangon Biotech (Shanghai, China).

**Table 1:** Sequences of nucleic acids used in this work.

**Name**	**Sequence (5′-3′)**
*S. aureus* (^a^D83356.1 ^b^192-237)	GGTTCAAAAGTGAAAGACGGTCTTGC TGTCACTTATAGATGGATCCGCGC
P1	GGTTCAAAAGTGAAAGACGGTCTTG
P2	GCGCGGATCCATCTATAAGTGAC

a *GenBank accession number*.

b *The position of specific sequence in genomic DNA*.

*The dotted line sequence of target was the same with primer P1. The underlined portion was complementary to the sequence of primer P2*.

### Extraction of Genomic DNA and RNA From *S. aureus*

The genomic DNA of *S. aureus* was extracted with TIANamp bacteria DNA kits (Tiangen Biotech, Beijing, China) according to the literature (Ulrich and Hughes, 2010). The target for colorimetric assay, specificity and anti-interference experiments. After enrichment culture for 24 h at 37°C, 1 mL bacterial liquid was heated at 95°C for 5 min, then centrifugated. One micro liter supernatant was used as target for colorimetric assay, specificity and anti-interference experiments.

### Isothermal Amplification Reaction

The SEA reaction was performed according to the manufacturers' instructions under optimal conditions. The fluorescence signal of the SEA reaction was detected by the Gentier 48S Isothermal amplification fluorescence detection system at 1 min intervals. Gel images were recorded by the ChampGel 5,000 system (Saizhi Innovation Technology Co. Ltd, Beijing, China). The reaction temperature was optimized at 57°C, 58.2°C, 60.5°C, 62°C, 63.5°C, 65.3°C, 66.6°C, and 67°C, respectively.

### Feasibility and Sensitivity of SEA Detection Method

The genomic DNA of *S. aureus* was used to demonstrate the feasibility of the SEA method. The sensitivity of the SEA detection method for *S. aureus* was evaluated with different dilutions of genomic DNA and DNA fragments. The culture fluids of *S. aureus* with *L. monocytogenes, S. typhimurium, V. parahemolyticus, S. castellani*, and *E. coli* were used to demonstrate the specificity and anti-jamming capacity of the SEA method.

### Colorimetric Assay by SEA Method

Colorimetric kits were obtained from Qingdao Navid Biotechnology Co. Ltd. (China). Rapid nucleic acid extraction from bacterial fluids was used to demonstrate the feasibility of SEA colorimetric method.

### Detection of *S. aureus* in Real Samples

The beef, pork, chicken, dried fish, and ham sausage real samples were collected from the small farmers markets (Qingdao, China). Samples were divided into 25 g each with a sterile knife. Then each sample was transferred to 225 mL 7.5% NaCl and then treated according to the literature with slight modifications (Abdalhai et al., 2014). After enrichment at 37°C for 24 h, 1 mL enrichment solution was taken from each sample for SEA and traditional plating detection, respectively.

## Results and Discussion

### The Design of SEA for the Detection of *S. aureus*

SEA was based on the single-stranded denaturation bubbles of dsDNA at the reaction temperature, including a pair of primers, *Bst* DNA polymerase, and a constant temperature. The specific primers (P1 and P2) were designed with 50 bp amplification fragment in the hypervariable V2 region of 16S rDNA (Table 1), which has a high copy number in bacteria. Briefly, one pair of specific primers bound with the targeted DNA fragments by invading the denaturing bubbles and induced DNA polymerase to extend the chain. In addition, according to the Tm values of primers, the reaction temperature was optimized using the SEA kit with *S. aureus* genomic DNA as the template. Finally, 62°C was chosen as the optimal reaction temperature (Figure S1) in this work.

### The Feasibility of SEA Method to Detect *S. aureus*

**Figure 1:**
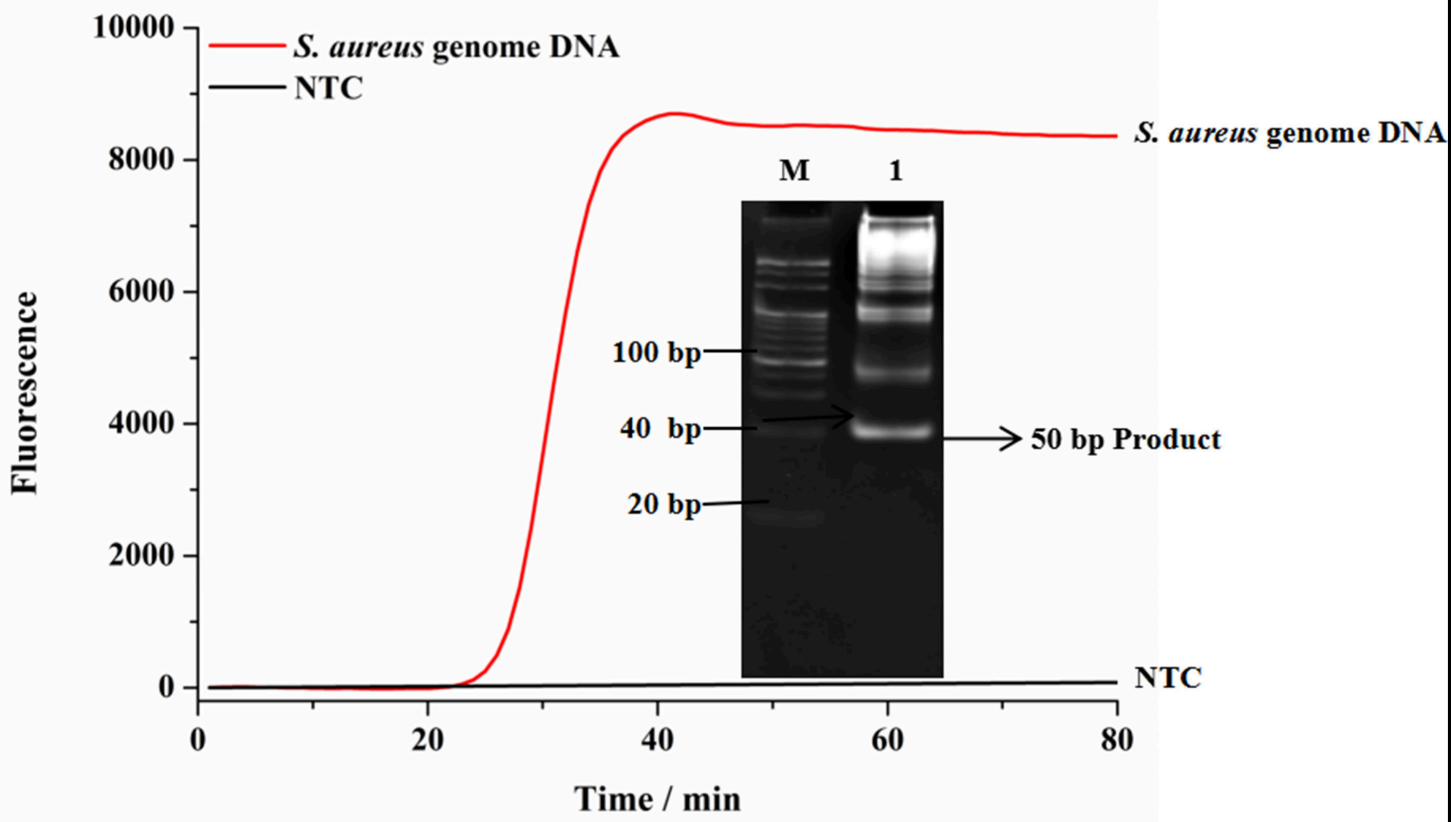
The feasibility of SEA to detect *S. aureus*. The red line represented that the targets were 1.0 × 10^−11^ M genomic DNA; The black line represented no targets control (NTC); Inset represented the amplified products in the gel electrophoresis was 50 bp.

Genomic DNA of *S. aureus* was used as the template to demonstrate the feasibility of SEA method to detect *S. aureus*. As shown in Figure 1, compared with the no target control (NTC) group, the fluorescence signal with the addition of genomic DNA significantly increased, which indicated that the SEA method could effectively detect genomic DNA of *S. aureus*. This result was consistent with the targeted 50 bp amplification products in the gel electrophoresis (Figure 1 inserted), which further confirmed the feasibility of SEA method to detect *S. aureus*.

### Sensitivity of SEA to Detect DNA Fragments and Genomic DNA of *S. aureus*

**Figure 2:**
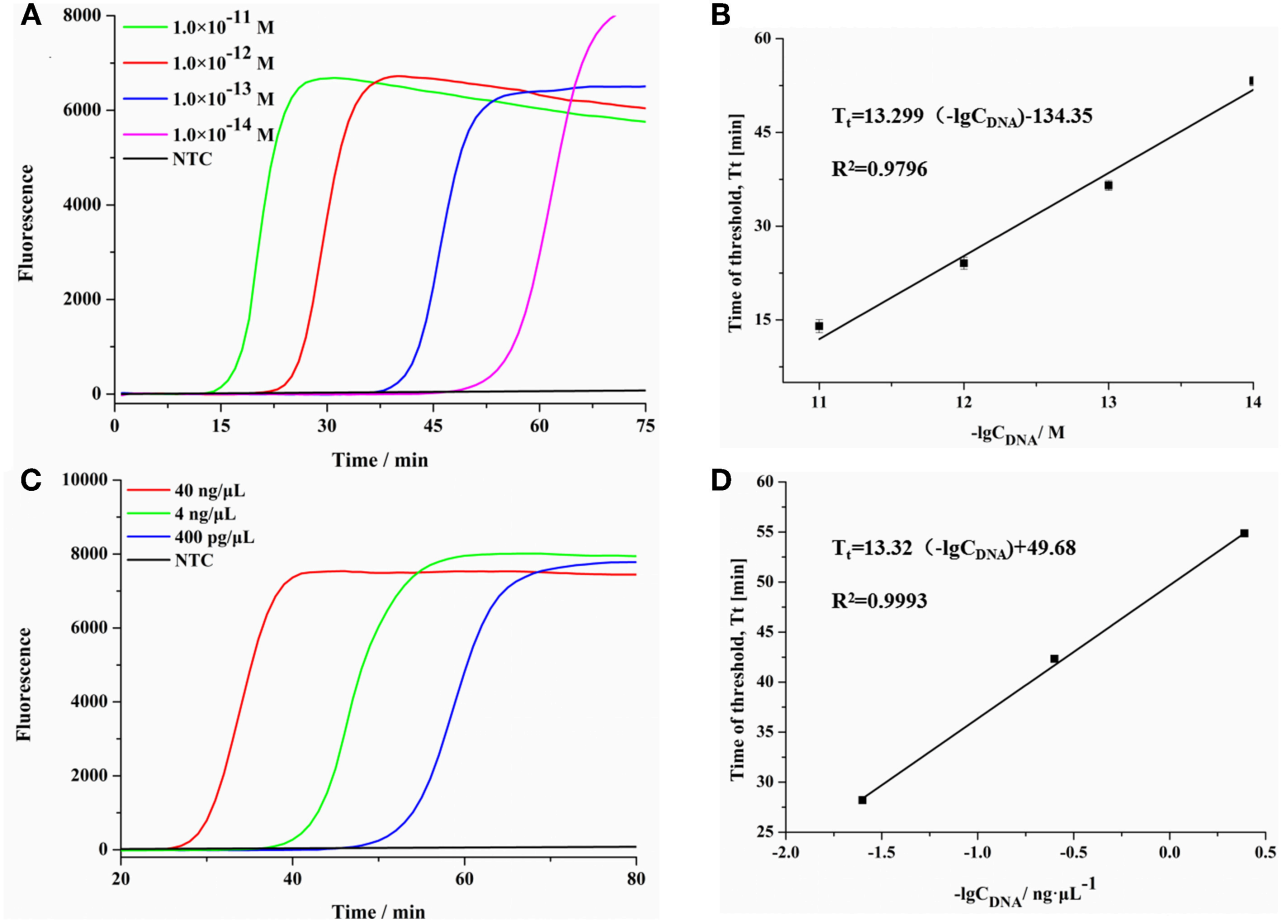
The sensitivity of SEA method for *S. aureus*. **(A)** The real-time fluorescence curves for different concentrations of *S. aureus* DNA fragment. **(B)** Relationship between the Tt values and the negative logarithmic values of the concentration of *S. aureus* DNA targets (DNA fragments concentration). Error bars showed mean standard deviations of three determinations. **(C)** The real-time fluorescence curves for different concentrations of *S. aureus* genomic DNA. **(D)** Relationship between the Tt values and the negative logarithmic values of the concentration of *S. aureus* DNA targets (DNA concentration). Error bars showed mean standard deviations of three determinations.

To evaluate the sensitivity of SEA detection method for *S. aureus* (Zhang et al., 2018), different concentrations of DNA fragments and genomic DNA of *S. aureus* were detected. As shown in Figure 2A, the fluorescence signals gradually increased with the increasing concentrations of targeted DNA fragments ranging from 1.0 × 10^−11^ M to 1.0 × 10^−14^ M. In addition, as shown in Figure 2B, the threshold time (Tt) value increased linearly with the increasing negative logarithm (lg) value of concentrations of *S. aureus* DNA fragments, ranging from 1.0 × 10^−11^ M to 1.0 × 10^−14^ M. The regression equation was Tt = 13.299 (-lgC_DNA_)−134.35 (C_DNA_ was the concentration of *S. aureus* DNA fragments, *R*^2^ = 0.9796). Moreover, SEA was also carried out using 10-fold serial dilutions of genomic DNA extracted from *S. aureus*. The results showed that SEA could be used to detect the concentration of *S. aureus* genomic DNA as low as 400 pg/μL (Figure 2C). As shown in Figure 2D, the Tt value increased linearly with the increasing lg of *S. aureus* genomic DNA concentrations in the range from 40 ng/μL to 400 pg/μL, which yielded a correlation equation of Tt = 13.32 (-lgC_DNA_)+49.68 (C_DNA_ was the concentration of *S. aureus* genomic DNA, *R*^2^ = 0.9993). In conclusion, the SEA method showed good linearity and sensitivity both in detecting genome DNA and DNA fragments.

### SEA Detection for Viable *S. aureus*

**Figure 3:**
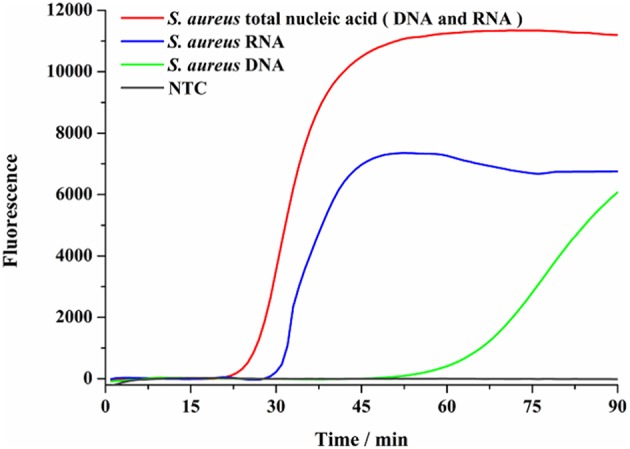
SEA detection on nucleic acids. Total nucleic acid was conducted to SEA detection of *S. aureus* (red line). The total nucleic acid was treated with DNA scavenger of recombinant DNase I before SEA detection of *S. aureus* (blue line). The total nucleic acid was treated with RNA scavenger of RNA-Be-Gone before SEA detection of *S. aureus* (green line).

RNA is readily degradable in the environment of pathogens *in vitro*, a biomarker of live bacteria, has been considered as a more suitable target for live bacteria detection. Therefore, using the SEA method to detect RNA and DNA in one step can not only take advantage of the abundance of RNA in live bacteria, but also make up for the low sensitivity of DNA detection. In this assay, total *S. aureus* nucleic acid (DNA and RNA), DNA and RNA were used as targets, respectively, to elucidate the nucleic acid detection efficiency of the SEA method. As shown in Figure 3, the SEA method could realize the detection of viable *S. aureus* by one-step detection of RNA by the fluorescence signal. We also found that the occurrence of the fluorescence signal was significantly delayed with DNA or RNA elimination, which indicated that detecting RNA and DNA simultaneously with the SEA method showed higher sensitivity than that of DNA or RNA independently. This result further verified that SEA method can not only detect DNA, but also RNA by one-step.

In addition, the targeted RNA fragments are relatively short in the SEA assay, so that both RNA and most of its incompletely-degraded RNA fragments could be used as amplification templates. This would undoubtedly improve the stability and sensitivity of SEA method for RNA detection.

### Detection of *S. aureus* by the Colorimetric Assay

**Figure 4:**
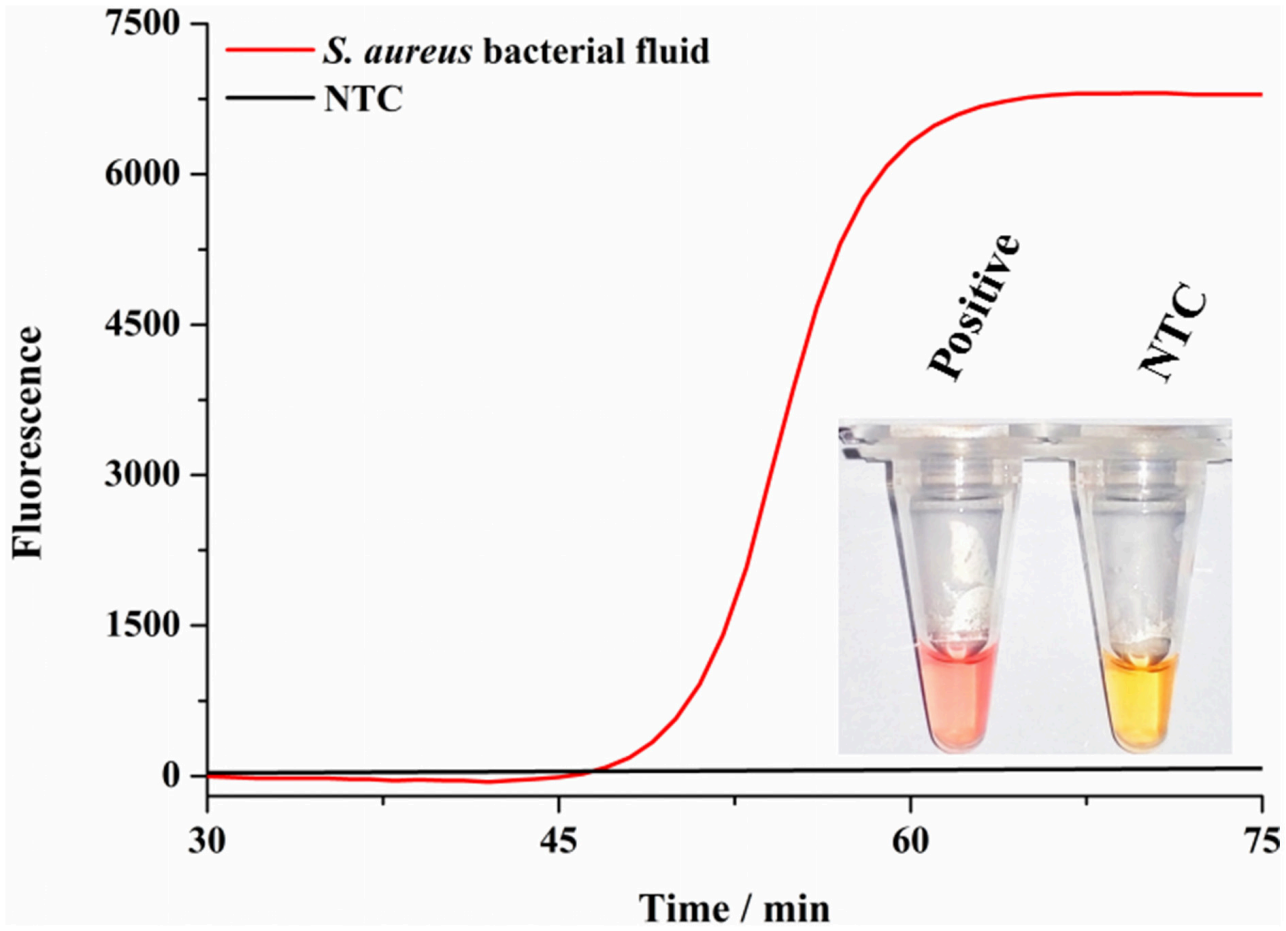
Detection of *S. aureus* by colorimetry. Fluorescence curves of the SEA reaction for *S. aureus* culture fluids and NTC; Inset represented the corresponding colorimetric result of SEA method to detect culture fluids of *S. aureus* and NTC.

Considering the wide existence in nature and severe effects of *S. aureus*, we have to make surveillance on it in various foods to ensure our health. At present, most rapid detection methods of *S. aureus* depend on complex and large-scale precision instruments, so that they are not suitable for developing regions. Therefore, it is necessary to develop a simple and rapid detection method for *S. aureus*. We developed a colorimetric method combined with SEA to detect *S. aureus*. As shown in Figure 4, the reaction mixture color could change from light yellow to red for positive samples and stayed light yellow for negative samples. Most importantly, the whole colorimetric detection procedure took no more than 50 min and was read out by the naked eyes, which was extremely simple and convenient. As shown in Figure 4 inset, the positive group changed its color from light yellow to red, while the NTC group remained the original yellow, which was consistent with the fluorescence results (Figure 4). Consequently, colorimetric detection results of the SEA method could be readily observed by the naked eyes instead of using the large and costly fluorescence detection equipment (Wang et al., 2014). In a word, the colorimetric SEA results could be performed without any complicated detection instruments or well-trained staff, which was especially applicable for the field detection of *S. aureus* in resource-limited environments.

### Detection of *S. aureus* in Real Samples

**Figure 5:**
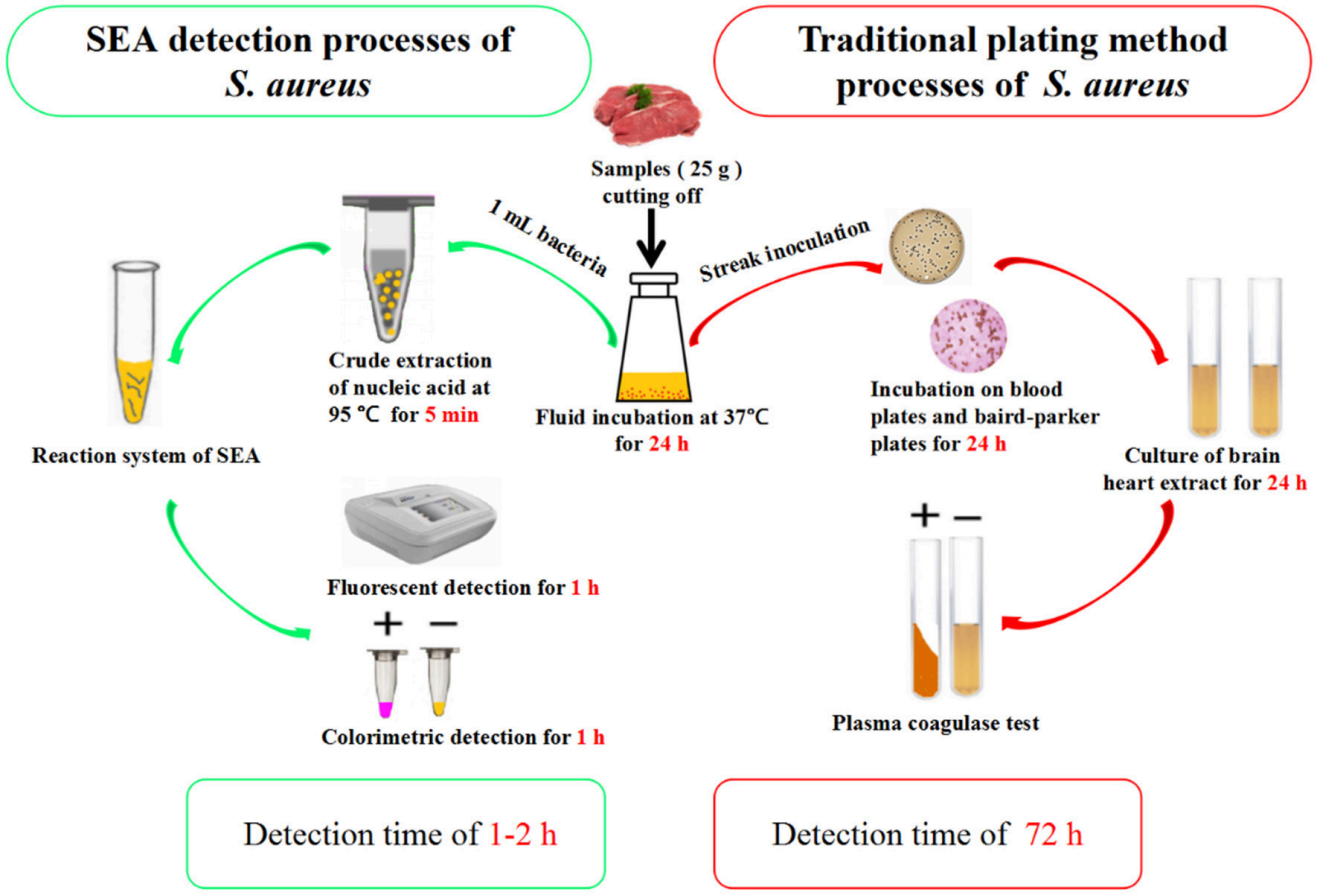
Comparison between SEA and traditional plating method for the detection of *S. aureus*.

Five other bacterial fluids, including *L. monocytogenes, S. typhimurium, S. castellani, V. parahemolyticus*, and *E. coli* were used to verify the specificity and anti-interference of the SEA method (Yang et al., 2016). As shown in Figure S2, the fluorescence signal only occurred in detecting *S. aureus* and no obvious effects were observed with the existence of other bacterial mixture, showing the good specificity and anti-interference of the SEA method. Thereafter, we carried out further artificially *S. aureus*-contaminated pork tests. The results showed that the detection limit of the SEA method on *S. aureus* was 10^0^ cfu/g (Figure S3), which met the requirements that *S. aureus* must not be detected out in foods. Furthermore, different meat real samples collected from the small farmers market were detected by the traditional plating method and SEA method for *S. aureus* simultaneously. As shown in Figure 5, the SEA method has a great advantage over the traditional plating method in detection time. The SEA method took only 1–2 h to detect real samples simply and rapidly, however, the traditional plating method need to spend as long as 72 h. In addition, positive rates of the SEA detection (Table 2) for *S. aureus* among those samples were consistent with that of the traditional plating method.

**Table 2:** Comparison of the detection of *S. aureus* by the SEA with the traditional plating method.

**Different detection methods**	**Number of samples**					
	**Total number of samples**	**Beef (27)**	**Pork (18)**	**Chicken (21)**	**Dried fish (17)**	**Ham sausage (29)**
SEA	Positive	3	2	2	1	1
	Positive rate	(11.1%)	(11.1%)	(9.5%)	(5.8%)	(3.4%)
The traditional plating methods	Positive	3	2	2	1	1
	Positive rate	(11.1%)	(11.1%)	(9.5%)	(5.8%)	(3.4%)

## Conclusions

A rapid and simple SEA method for the detection of *S. aureus* was developed in this work. SEA method to detect RNA of *S. aureus* by one step took advantage of the high abundance of RNA in live bacteria, which made SEA method not only able to detect viable *S. aureus*, but also to greatly improve the low sensitivity of DNA detection. Furthermore, the colorimetric detection could be conducted at a constant temperature and then read out results by the naked eyes. The whole SEA detection procedure for real samples took only 1–2 h, which was so time-saving compared with the traditional plating method taking as long as 72 h. Thus, the SEA method provided a simple, rapid and equipment-free detection platform. It was also expected to be incorporated into microfluidic chips to realize the sample-to-answer diagnostic in a single device for further POCT purpose.

## Author Contributions

CS conceived and designed the experiments. CL performed the experiments. ML and MW analyzed the data. CM and ZW wrote the paper. CS revised and approved the final version.

### Conflict of Interest Statement

The authors declare that the research was conducted in the absence of any commercial or financial relationships that could be construed as a potential conflict of interest.

## Abbreviations

*S. aureus*: *Staphylococcus aureus*
SEA: strand exchange amplification
POCT: point-of-care testing
PCR: polymerase chain reaction
ELISA: enzyme linked immunosorbent assay
NASBA: nucleic acid sequence-based amplification
LAMP: loop-mediated isothermal amplification
SDS: sodium dodecyl sulfate
HPLC: high performance liquid chromatography
NTC: no target control
PAGE: polyacrylamide gel electrophoresis.
