# Supplementary material for: Rapid and Simple Detection of Viable Foodborne Pathogen Staphylococcus aureus
Source: Front Chem. 2019 Mar 12;7:124. doi: 10.3389/fchem.2019.00124 (PMC6424009; doi:10.3389/fchem.2019.00124)
Supplement: Supplementary file 1 [file Data_Sheet_1.doc]

Supplementary Material

**Rapid and simple detection of viable foodborne pathogen *Staphylococcus aureus***

**Caiyan Liu^1†^, Chao Shi^1†^, Mengzhe Li^1^, Mengyuan Wang^2^, Cuiping Ma^3^ and**

**Zonghua Wang^1*^**

*1 Shandong Sino-Japanese Center for Collaborative Research of Carbon Nanomaterials, College of Chemistry and Chemical Engineering, College of Life Sciences, Qingdao University, Qingdao, China,
2 The Affiliated Hospital of Qingdao University Medical College, Qingdao, China,*

*3 Shandong Provincial Key Laboratory of Biochemical Engineering, College of Marine Science and Biological Engineering, Qingdao University of Science and Technology, Qingdao, China*

*** Correspondence:**

Zonghua Wang

[**wangzonghua@qdu.edu.cn**](mailto:wangzonghua@qdu.edu.cn)

| 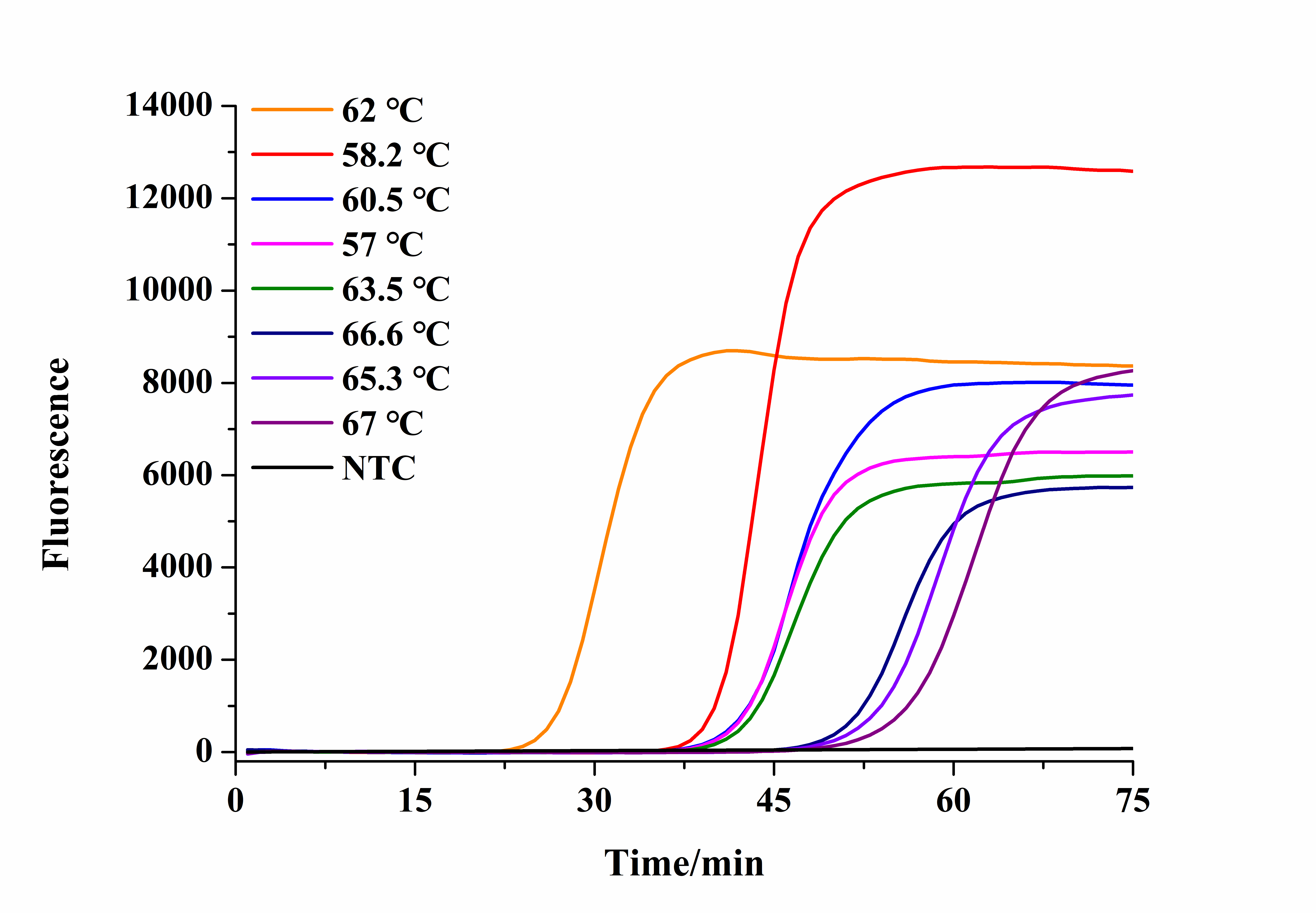  **Figure S1.** The optimization of reaction temperature of SEA to detect *S. aureus.* |
| --- |


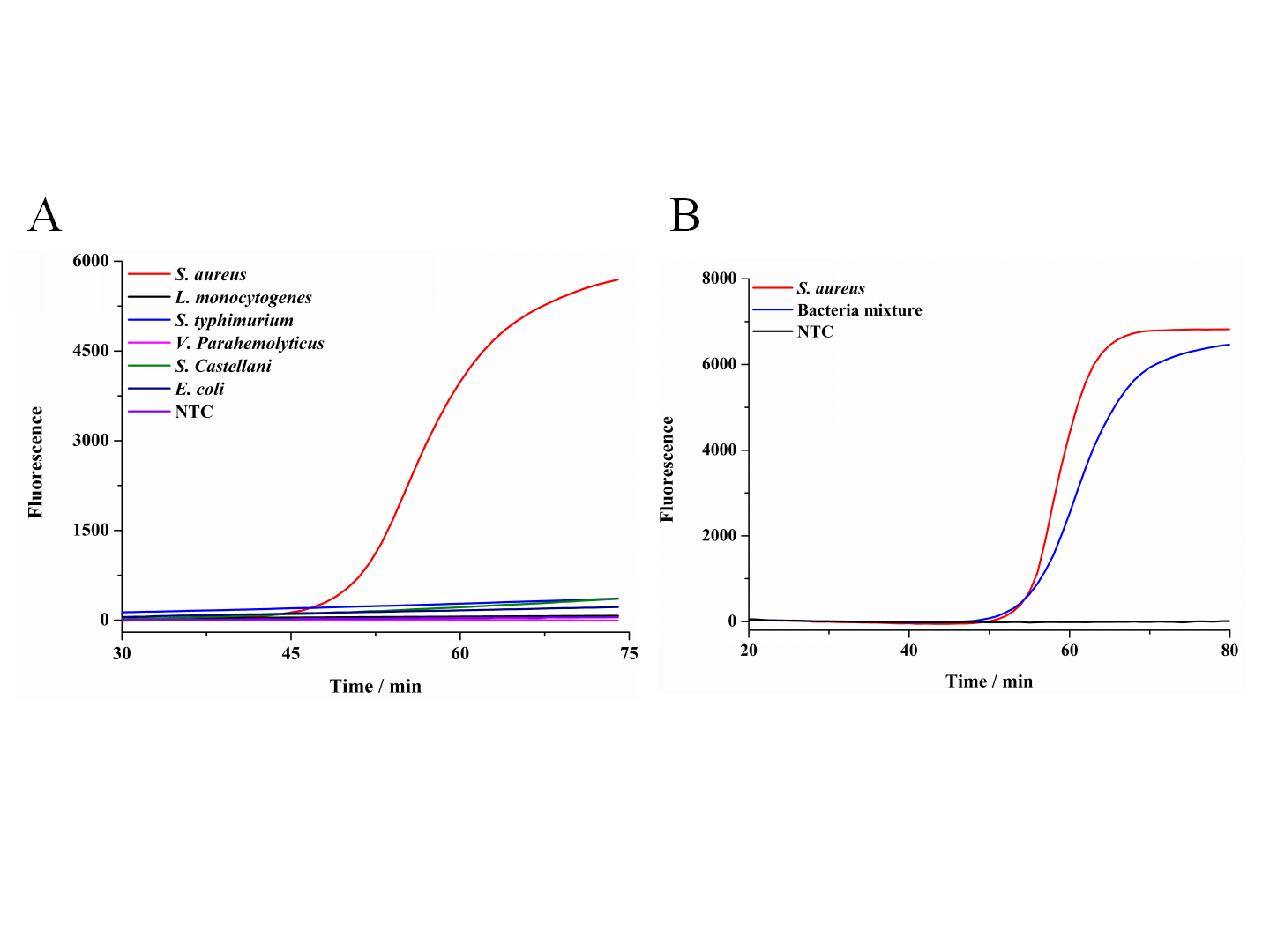


**Figure S2.** The specificity and anti-jamming of SEA detection method for *S. aureus.* (A) Specificity of the SEA method for detecting the culture fluids of *S. aureus* with *L. monocytogenes, S. typhimurium, V. Parahemolyticus, S. Castellani* and *E. coli.* (B) Anti-jamming capacity of the SEA method. The red line represented that the targets were the culture fluids of *S. aureus* diluted 10-fold. The blue line represented that the targets were the mixture of the culture fluids of *S. aureus*, *L. monocytogenes*, *S. typhimurium*, *V. Parahemolyticus*, *S. Castellani* and *E. coli* diluted 10-fold with the ratio of 1:1:1:1:1:1; the black line represented the NTC.


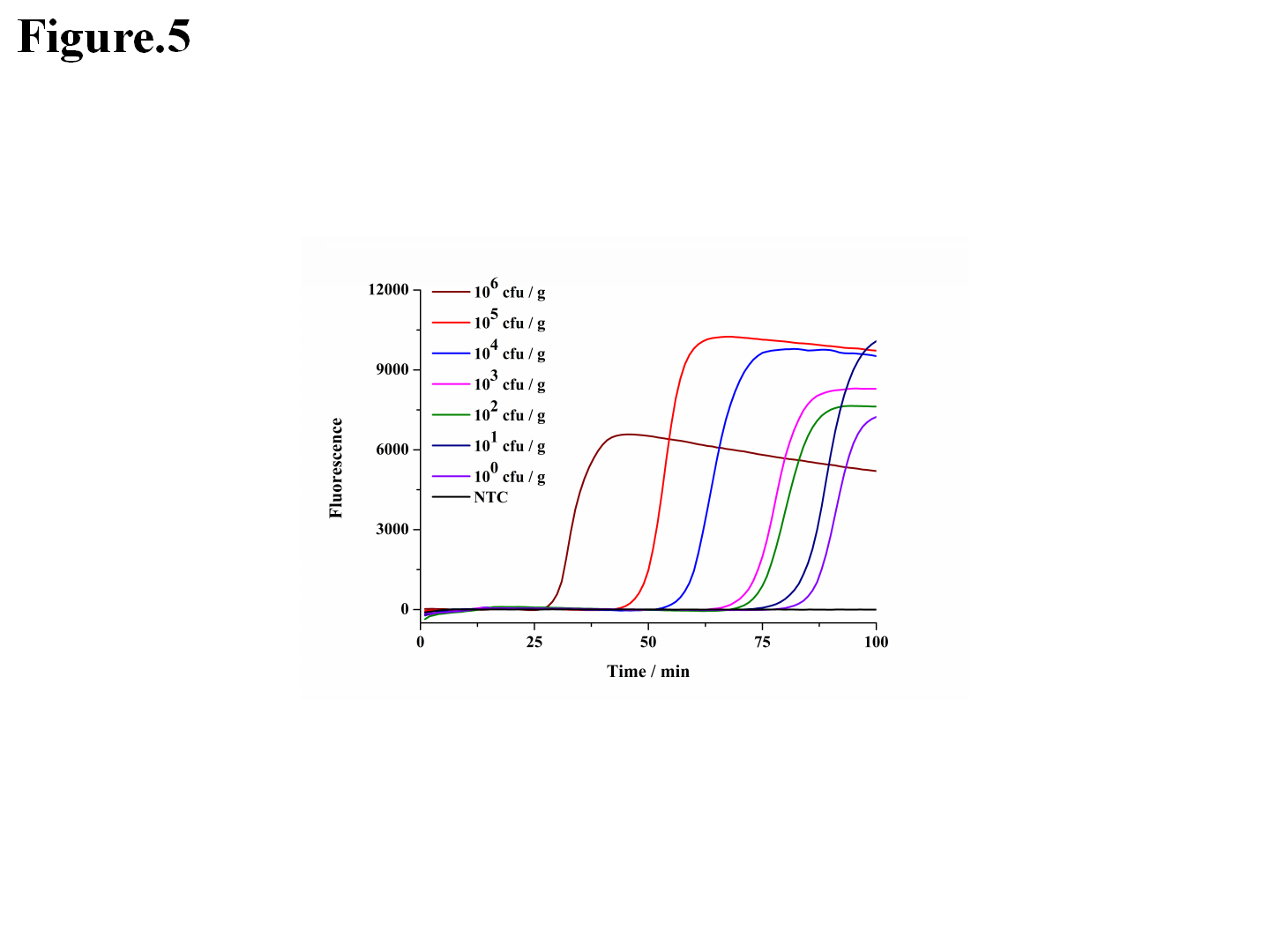


**Figure S3**. Detection *S. aureus* of artificial contaminated pork meat by SEA method. The real-time fluorescence curves of *S. aureus* from 1.0 × 10^6^ to 1.0 × 10^0^ cfu/mL were spiked into pork, respectively.
